# Supplementary material for: Torquetenovirus in saliva: A potential biomarker for SARS-CoV-2 infection?
Source: PLoS One. 2021 Aug 24;16(8):e0256357. doi: 10.1371/journal.pone.0256357 (PMC8384193; doi:10.1371/journal.pone.0256357)
Supplement: Attachment — Submitted filename: PLoS One response to reviewers_03_05_21.docx [file pone.0256357.s001.docx]

Baochuan Lin, Ph.D. Academic Editor, PLOS ONE - PONE-D-21-05788R1

Dear Dr. Lin,

We thank you and the reviewers for the positive and constructive comments on our manuscript. The individual comments and our responses are listed below.

**Reviewer #1: Thanks for the opportunity to review the manuscript. The research idea was novel and attractive. However, the title is not completely compatible with the data and the methods that was provided in the manuscript. I have some comments and questions:**

**Answer:** The title has been revised and now reads: Torquetenovirus in saliva: a potential biomarker for SARS-CoV-2 infection?

**1- About the SARS-CoV-2 sampling, as I found the patients were instructed and did the sampling by themselves, using nasopharyngeal swabs. This method (nasopharyngeal swabs) is a sensitive sampling method but is painful and disgusting. How could you be sure that samples were collected correctly? I mean, how could you be sure that the negative samples were not false negative results?**

**Answer:** As stated in the text, subjects were given a detailed video that clearly demonstrated the correct way to collect samples. Follow-up of subjects confirmed that all negative assays were truly negative. We included the term “nasal-oropharyngeal swab sample” in order to clarify the appropriated procedure which we used in this study.

**2- We are in the pandemic of COVID-19, could we assume a symptomatic individual as a control just with a single SARS-CoV-2 negative PCR?**

**Answer:** As mentioned above we have had follow-up contact with all negative controls to verify that they were not infected. We also included a sentence in the lines 111 to 113 to clarify such aspect.

**3- It was a good idea to select the controls among symptomatic individuals who had negative SARS-CoV-2 PCR, but we need more information about the cause of disease in controls because an allergic rhinitis would be different from common cold. Moreover, some of the controls could have other strains of corona virus. I afraid the controls are not homogenous.**

**Answer:** We thank the reviewer for this comment that requires clarification. The totality of symptoms in each of the controls were characteristic of the common cold. Those with allergic rhinitis were excluded. This is now stated in Methods (in the lines 85 to 86). We did not test for the presence of other coronavirus strains in the controls. Their presence was unlikely since the prevalence of these viruses is rare in our population.

**4- In line 97, you wrote “Several individuals positive for SARS-CoV-2 in their saliva were requested to collect additional saliva samples over a 30-day period.”, what about individuals with negative PCR?**

**Answer:** We only collected additional samples from SARS-CoV-2-positive patients. It probably would have been optimal to also collect additional samples from the controls. However, it was apparent that there was no clinical indication of symptom worsening or the presence of Covid in this group.

**5- About the clinical sign and symptoms, did the patient measured his/her body temperature? If yes, is this a sensitive method to divide patients to febrile and afebrile?**

**Answer:** Body temperature was self-collected and, we agree, may not always be accurate. However, this was not the only parameter evaluated.

**6- Could we have more information about the drug history and background disease of both cases and controls? Because it might affect the TTV viral load.**

**Answer:** We have now added to the text in Methods that none of the subjects used antibiotics or other medications in the two weeks prior to sample collection (in the lines 87 and 88). In addition, they were all healthy with no underlying pathology.

**7- In table 3, you showed that the SARS-CoV-2-positive patients had the highest median TTV viral load on day 8-9 while the viral load was lower in day 6-7, how could you interpret this finding considering the normal immunologic response to SARS-CoV-2 infection?**

**Answer:** The viral loads on days 6-7 and 8-9 are very similar and statistically indistinguishable.

**8- Could you describe, why you divided the study course to days 3-5, 6-7, 8-9, 10-16,…? Why the intervals are not equal?**

**Answer:** The interval was chosen to have approximately the same number of subjects at each time point. We believed that this provided the most reliable indication of changes.

**9- You only provided data about the kinetics of SARS-CoV-2 virus, what about the clinical course of disease in different time points, data on hospital admission, or mortality?**

As stated in the text, this is a study of patients with mild symptoms that resolved during the study period. None required hospitalization admission.

**10- About the figure 1, this is a spaghetti plot showing the kinetics of TTV, if each shape is representative for a single patient, I afraid you had a lot of missing data. Could you provide the figure legend with more details about the plot and shapes?**

**Answer:** The figure legend has now been implemented to state more explicitly that the graph measures the TTV titer in saliva at each time point for each subject where more than one sample was obtained (in the lines 213 to 216).

**11- You might need to provide the immunologic markers, as well as more information about the clinical course of disease, if you aim to conclude that TTV is a marker of local immune status and as predictors of prognosis.**

**Answer:** Immune markers were not measured in these subjects and so we only speculate that the reduction in TTV as a predictor of prognosis occurred at least partially by a mechanism of immune activation. This was based on other studies relating TTV titer to immune system status. We have now clarified in Discussion that an immune-mediated mechanism was not directly tested and that our findings are consistent with an increased overall immune competence (in the lines 252 and 253).

**Reviewer #2: In this manuscript, Mendes-Correa et al. focuse on the attempt to demonstrate that TTV level in saliva from COVID-19 patients might be used as biomarker for progression or resolution of SARS CoV-2 infection. Although the topic may be of interest, the way it is treated is disappointing and a number of issues makes weak the manuscript. Overall, in the current form, the manuscript does not reach the quality standard needed for publication**

**Answer:** We appreciate the reviewer’s concerns and trust that our responses to the specific comments pf the other reviewers will alleviate the reviewer’s concerns.

**Reviewer #3: Interesting study from a single center cohort about the association of TTV titer in saliva with SARS-CoV-2 status.**
**1) the major limitation is already discussed in the manuscript:
the SARS-CoV-2 negative group - all with "mild respiratory symptoms" was not further characterized.
Especially testing with a multiplex-PCR for other respiratory viruses would help to interpret the data in the manuscript, especially since TTV levels seem to stay unchanged in the SARS-CoV-negative group day 10-16 and >16 in contrast to the SARS-CoV-2 positive group.
If saliva samples are still available, I would recommend performing these analyses in both groups.
The manuscript could be further improved if a matched control group without respiratory symptoms would also be tested for TTV in saliva.**

**Answer:** Unfortunately, the subjects in this study were not tested for other respiratory viruses and the samples are no longer available for this purpose. As the reviewer states, and as we acknowledged in Discussion, this as a study limitation. While it would certainly be of interest to determine the prevalence and titer of TTV in healthy individuals, and this is planned for the near future, that information is not essential for data analysis in the present investigation.

**2) line 49: it is true that TTV has been described as an indicator of immune function mainly after organ transplantation, but there is also literature on TTV as a marker of immune function in chronic diseases such as liver cirrhosis or HIV. This could be briefly elaborated by the authors to show that looking at TTV levels could also have a value outside of organ transplantation.**

**Answer:** We thank the reviewer for this suggestion and have now added the additional information to Introduction with references (in the lines 55 to 57).

**3) line 53/54: should be probably ref. 6 (TTV in HSCT recip.); "predicting lymphocyte status" is somewhat obscure to me, would suggest immune function, immunocompetence or immunreconstitution.**

**Answer:** The text has been changed as suggested to now read “immunocompetence” and reference 8 has been added (in the line 61).

**4) line 191: it would be helpful to provide the number of subjects with more than one saliva sample, as this is rather small and therefore a cautious interpretation is required**

**Answer:** The number of subjects with more than one saliva sample is evident from Figure 1. We have now added this, 10 subjects, to the text as suggested (in the lines 213 to 216).

**5) line 203: would remove "anti-SARS-CoV-2" as no rationale is provided that TTV level reflects specific anti-SARS-CoV-2 immune activity - considering the literature, it is more likely a marker of overall immunocompetence**

**Answer:** We agree, and has been stated in comment 11 for Reviewer #1, a mechanism revolving around altered immune status was speculative and not based on collection of immune-related parameters. The term “anti-SARS-CoV-2” has been deleted.

**Reviewer #4: The authors describe the evolution of torque tenovirus viral load in saliva related to , 10Sars-CoV 2 viral load in nasopharyngeal swabs, in an effort to find markers for immunosuppression. They also compare TTV levels at different times post initiation of symptoms for the covid 19 population (mild covid) and a control population with a mild respiratory disease.**

**Second, the authors show in a subpopulation of the covid 19 group that TTV viral load decreases with the disparition of symptoms. As a consequence, they consider that TTV viral load in saliva may be suitable to describe rhinopharyngeal local immune response. And they refer to studies comparing blood TTV viral load and evolution of immunity /rejection in transplant patients.

The idea is interesting, because simple tests to evaluate local immunity are very important to identify potential patients that could evolve toward high viral loads and covid worsening. The data concerning the evolution of TTV viral load in relation to SARS-CoV 2 viral load is convincing. This is, to my knowledge, the first paper describing TTV load in saliva for covid patients .**

**Answer:** We thank the Reviewer for acknowledging our unique hypothesis regarding TTV analysis in Covid patients and its eventual clinical potential.

**Although, the paper suffers from some weaknesses that could be overcome:

First : the authors did not justify the use of saliva instead of the Nasopharyngeal samples for TTV viral load. Although they refer to its use in previous comparisons with plasma levels in transplant patients to predict global immunity which is very different. They use the same extraction method . We understand of course that saliva is easier and potentially more reproducible for auto collection, but the TTV load values, in saliva, are highly variable in both populations and as NOP sampling was performed, they could have compared TTV load in both samples, to comfort their hypothesis.**

**Answer:** We thank the reviewer for this comment. Unfortunately, the NOP samples were not available for TTV testing when the study was designed.

**Second : The large panel of viral loads at each time, coming from the fact that there was only one sample per patient and thus at each time we compare different patients make the group comparison less significant, the authors should comment on this and provide a graph in addition to the table to show the repartition of viral loads within time in both groups.**

**Answer:** We agree with the Reviewer that the use of saliva from different subjects at different time points is not optimal, and this is acknowledged in the Discussion as a study limitation as requested. We disagree with the Reviewer that in addition to the Table we should also provide a graph. We think this is unnecessarily repetitious and does not provide any additional information.

**Third : The lowering of TTV viral load from sequential samples in the covid patients cannot be compared with that of the non-covid group. In addition we do onot have the diagnosis (VRS may be different from rhinovirus for example in terms of local immunity, samely pharyngitis or thracheitis may be different from coryza?) Theu could add the diagnosis if available.**

**Answer:** We responded to similar comments by Reviewer 1, comment #3 and Reviewer #3, comment 1 and agree that the control group might possibly be heterogeneous. We did not test the control subjects for the possible presence of different rhinoviruses as acknowledged as a study limitation in Discussion. Based solely on their symptoms and recovery time without treatment it appeared most likely that they had the common cold, and not an allergic rhinitis or a bacterial pharyngitis or tracheitis. We have now added a comment in Methods to further clarify this limitation.

**Fourth : The authors have choosen a mild covid group for a better comparison with mild respiratory disease. Could they correlate the initial TTV load or the maximum TTV load , with the duration of symptoms , or the delay to SARS-CoV 2 undetectability? This could be very interesting to predict persistent infections.**

**Answer:** We thank the Reviewer for this very constructive suggestion. Unfortunately, in our study these additional comparisons were not possible. Our sample size was too small to perform this analysis with a high degree of validity.


**Minor comments : some text errors or form errors are present in the text and bibliography, and a cautious review is necessary before submission.**

**Answer:** We apologize for these errors and have now corrected them in the revision.

We are available to send you any further information you may require.

Yours faithfully,

Tania Regina Tozetto-Mendoza and co-authors.
