# Supplementary material for: Torquetenovirus in saliva: A potential biomarker for SARS-CoV-2 infection?
Source: PLoS One. 2021 Aug 24;16(8):e0256357. doi: 10.1371/journal.pone.0256357 (PMC8384193; doi:10.1371/journal.pone.0256357)
Supplement: Attachment — Submitted filename: RESPONSE TO REVIEWERS_D-21-05788R1_PLoS One comments.docx [file pone.0256357.s002.docx]

RESPONSE TO REVIEWERS

Baochuan Lin, Ph.D.
Academic Editor
PLOS ONE

Dear Dr. Lin:

Thank you and the reviewers for the responses to our revised manuscript, PONE-D-21-05788R1, “Torquetenovirus in saliva: a potential biomarker for SARS-CoV-2 infection?” The remaining reviewer comments (underlined) and our responses are listed below.

**Reviewer #1**

About the response to comment 3, you responded, "We did not test for the presence of other coronavirus strains in the controls. Their presence was unlikely since the prevalence of these viruses is rare in our population.". As I can see in literature, prevalence of human coronaviruses (HCoVs) is about 11.5% ([https://doi.org/10.1590/S1517-83822013000100049](https://urldefense.proofpoint.com/v2/url?u=https-3A__doi.org_10.1590_S1517-2D83822013000100049&d=DwMFaQ&c=lb62iw4YL4RFalcE2hQUQealT9-RXrryqt9KZX2qu2s&r=GkVeazppdM1GOrt42pvg0z0w4N7OfqswaK2i1TXhaQ4&m=33n0OicZ_I_5mOq21xVAvxMXEvt2oFv4yeWeqtSXkJw&s=6ATC9ELoLQ6azb81v2fp1YPh_UNB_-HXxIUv9hEcDVA&e=)).

**Answer:** We appreciate the reviewer pointing out to us this relevant publication that we unfortunately had not previously seen. It is now cited in the revised text and included in the Reference section (reference 25, lines 341-343). The Discussion section has been extensively revised to more accurately reflect our observations and study limitations, as well as to bolster our argument that other viruses that may have been present in the nasopharynx likely did not have an appreciable influence on our findings. A portion of the revised Discussion is presented here: “Other viruses^24^, including different coronavirus species^25^, may also have been present in the nasopharynx of our study population. Thus, the possible influence of viral co-infections on SARS-C0V-2-related salivary TTV levels remains undetermined. We would stress, however, that the uniform responses in the SARS-CoV-2 positive and negative patients suggest that any influence of other co-infections is likely to be relatively minor. A parallel diminution in TTV titer over time after resolution of respiratory symptoms was not observed in the SARS-CoV-2-negative individuals. This strongly suggests that immune alterations associated with a mild respiratory tract infection are insufficient to have a noticeable influence on TTV levels in saliva”.

Although the kinetics of TTV could be a possible marker of local immunity, but considering your results, it is difficult to predict prognosis using TTV.

**Answer:** We agree with the reviewer that quantitation of TTV in saliva may not ultimately prove to be a sensitive indicator of SARS-CoV-2 prognosis. Our inclusion of a question mark at the end of the title of our manuscript signifies this possibility. It is our strong belief, however, that this possibility is worthy of further investigation and that publication of our observations will bring this possibility to the attention of the research community. We also believe that our revised Discussion further supports our argument that the kinetics of TTV is a valid maker of local immunity (in manuscript lines 228 to 334 and lines 250 to 260).

**Reviewer #3**

 While the lack of testing for other respiratory viruses and healthy controls somewhat limit the scientific value, the data should be still of interest for the scientific community.

**Answer:** We thank the reviewer for acknowledging the interest of our findings for the scientific community. Please see the comments to Reviewer #1 for our revisions that we believe further increase their scientific value.
