# Supplementary material for: Torquetenovirus in saliva: A potential biomarker for SARS-CoV-2 infection?
Source: PLoS One. 2021 Aug 24;16(8):e0256357. doi: 10.1371/journal.pone.0256357 (PMC8384193; doi:10.1371/journal.pone.0256357)
Supplement: Attachment — Submitted filename: RESPONSE TO REVIEWERS - PONE-D-21-05788R4.docx [file pone.0256357.s004.docx]

RESPONSE TO REVIEWERS - PONE-D-21-05788R4

Baochuan Lin, Ph.D.

Academic Editor

PLOS ONE

Dear Editor,

Thank you for the specific comments to our revised manuscript, PONE-D-21-05788R3, “Torquetenovirus in saliva: a potential biomarker for SARS-CoV-2 infection?”

All changes were marked in blue color in the revised manuscript to each point raised by the academic editor. We apologize for these errors and have now corrected them in the revision.

Yours faithfully,

Tania Regina Tozetto-Mendoza, *Ph*D

*Laboratory of Virology,*

*Institute of Tropical Medicine*

*School of Medicine, University of São Paulo, Brazil*

*E-mail: tozetto@usp.br*

July, 30, 2021
